# Eating attitudes and trauma in third-generation holocaust survivors

**DOI:** 10.3389/fpsyg.2026.1718777

**Published:** 2026-05-07

**Authors:** Goni Biran, Lea Jakob

**Affiliations:** 1 Department of Psychology, University of New York in Prague, Prague, Czechia; 2 Department of Philosophy, Sociology, Education, and Applied Psychology, University of Padova, Padua, Italy

**Keywords:** controlling feeding, eating attitudes, feeding behaviors, holocaust trauma transmission, third-generation

## Abstract

This cross-sectional study examined the transgenerational transmission of Holocaust trauma on eating and food attitudes among third-generation Holocaust survivors. Limited knowledge exists on the nature of Holocaust exposure for the third-generation, especially concerning disordered eating. A convenience sample of 236 Israeli respondents aged 18–50 was recruited via Google Forms and was asked to complete the EAT-26 questionnaire and a custom questionnaire. Gender differences were observed, with women showing higher EAT-26 scores, indicating a less healthy relationship with food. The study included 130 third-generation (3rd-gen) and 106 non-third-generation (non-3rd-gen) participants. Although no significant differences were found between the groups in dieting, weight concerns, and EAT-26 scores, significant differences were discovered regarding their perception of parental feeding and education styles. It can be concluded from the results that Holocaust transmission of trauma persists in the third-generation, manifested through their relationship with their parents and the feeding style they experienced in their childhood.

## 1 Introduction

Transgenerational transmission of trauma refers to the process by which a traumatic event’s psychological and emotional consequences are passed on from one generation to another (Kellermann, 2001a; Kellermann, 2001b). This process can occur through various mechanisms, including genetic, epigenetic, and environmental factors (Yehuda and Bierer, 2009). Nearly eight decades have passed since the end of World War II and the Holocaust. The psychological consequences of the terrors experienced by the Jewish people during the Holocaust continue to affect the mental and physical health of second and third generations of Holocaust survivors (Shrira et al., 2011). The experience of being captive in the ghetto was marked by an absence of basic necessities for a healthy existence, with food rationing being the most striking feature, leading to severe hunger and starvation-caused mortality among the Jewish population (Massry and Smogorzewski, 2002).

Evidence for transgenerational effects of extreme trauma has also been documented in other historical contexts characterized by mass starvation and genocide. Studies of descendants of survivors of the Dutch Hunger Winter and the Leningrad Siege have demonstrated long-term alterations in health outcomes and eating-related patterns among subsequent generations (Tolkunova et al., 2023). Similarly, research on post-genocide populations, such as descendants of the Holodomor and the Rwandan genocide, indicates persistent intergenerational transmission of trauma-related behaviors and health vulnerabilities (Zasiekina et al., 2021; Kahn and Denov, 2022).

The experience of living in the ghetto has been shown to have severely damaged Holocaust survivor’s mental health and resulted in them having an altered, unhealthy relationship with food. Even though the Holocaust survivors’ children were not present when the parent went through the trauma, they feel like they went through the trauma themselves (Kellermann, 2001a; Kellermann, 2001b; Levav et al., 2007; Weiss and Weiss, 2000). There is a vast amount of evidence that supports that the second-generation of Holocaust survivors have been affected by their parent’s attitudes and ways of education regarding feeding. However, there is very little research regarding the transmission of trauma and eating attitudes among the third-generation of Holocaust survivors. This study proposes that the intergenerational transmission of Holocaust trauma from second-generation parents may manifest in their children, the third-generation, leading to unhealthy eating and attitudes toward food, thereby indicating that the effects of the trauma are transmitted across the generations.

### 1.1 Eating behaviors in holocaust survivors and feeding

Exposure to extreme hunger and starvation-related deaths during the Holocaust undeniably led to trauma in survivors. Therefore, many Holocaust survivors have post-traumatic stress disorder that also takes place with their attitude toward food (Sindler et al., 2004; Zohar et al., 2007). As parents serve as role models for their children, children often mimic their parents’ eating habits and attitudes toward food. Parents create the food environment in the home, such as the food available and the attitudes toward eating. Parents’ choices and parenting styles impact how children will consume food in the future and their positive or negative mindset toward eating (Scaglioni et al., 2018).

### 1.2 Eating behaviors of the second-generation

Chesler’s (2005) study highlights how Holocaust survivors’ trauma and deprivation impact their children’s eating behaviors, finding that all second-generation participants experienced ongoing or past eating disruptions. Participants had abnormal relationships with food, with some overeating and others restricting intake, driven by guilt, fear of weight gain, or pressure to consume everything. Children of concentration camp survivors, for instance, were encouraged to eat all food, reflecting a deep-rooted survival emphasis. Similar results were exhibited in Halioua et al.’s (2025) study in which children of Holocaust survivors were more likely to keep large food stores, eat quickly, eat all the food, and overeat than the comparison group. Additionally, restrained eaters often had parents who prioritized slimness and controlled eating. This dynamic may extend into the third generation, reflecting the persistent influence of intergenerational trauma on eating behaviors.

### 1.3 Eating behaviors of the third-generation

In a study by Zohar et al. (2007), females who are third-generation and their mothers who are second-generation Holocaust survivors were assessed to find a connection between the family’s Holocaust past and eating disorders. According to the study, there was a relationship between the student’s relationship with food, their mother’s eating disorders, and exposure to the Holocaust by grandparents. The link between mothers’ eating pathology and its effect on their daughters is likewise featured in Barnett et al. (2005) study, which supports that mothers’ low self-esteem and poor perception of their bodies will affect their daughters by having higher chances of developing an eating disorder. In addition, a study by Oren and Shavit (2024) revealed that Holocaust trauma persisted in the third-generation of Holocaust survivors in the form of avoidance of discarding food.

This study suggests that the transmission of Holocaust trauma will influence second-generation parents’ feeding practices they promote to the third-generation and therefore may exhibit in their controlling behaviors and approach to feeding. We hypothesize that (H1) third-generation individuals with Holocaust survivor grandparents will report a more disordered relationship with food compared to those with non-survivor grandparents. Secondly (H2), the degree of disordered eating behaviors will be positively correlated with the participants’ perception of their parents’ dysfunctional relationship with food, and this relationship would be stronger among 3rd-gen participants than non-3rd-gen participants. Lastly (H3), third-generation participants will view their parents’ feeding style and attitudes toward food as more controlling compared to participants with no Holocaust-related family history.

By focusing on eating behaviors in third-generation Holocaust survivors, this study extends existing research on transgenerational trauma and provides novel insight into how historical trauma may be transmitted through family feeding practices and attitudes toward food, testing three hypotheses. First, it was hypothesized that third-generation individuals with Holocaust survivor grandparents would report a more disordered relationship with food compared to individuals without Holocaust-related family history (H1). Second, it was hypothesized that greater disordered eating would be associated with participants’ perceptions of their parents’ dysfunctional relationship with food, and that this association would be stronger among third-generation participants than among non-third-generation participants (H2). Third, it was hypothesized that third-generation participants would perceive their parents’ feeding style and attitudes toward food as more controlling compared to participants with no Holocaust-related family history (H3).

## 2 Methods

### 2.1 Sample

The data collection methods included the Eating Attitude Test-26 (EAT-26). Additional questions were combined in the distributed form to explore relevant factors the EAT-26 lacks. The participants were gathered using convenience and snowball sampling.

### 2.2 Research design

#### 2.2.1 Measures

At the beginning of an online form, the participants were asked to provide sociodemographic information, including gender, age, city name, and if they are grandchildren of Holocaust survivors. Participants were classified as third-generation if they reported having at least one Holocaust survivor grandparent; participants reporting no Holocaust survivor grandparents were classified as non-third-generation.

The EAT-26 (Garner et al., 1982) is a self-report assesment and was used to measure eating attitudes and behaviors. It is rated on a six-point Likert scale and comprises of three factors: (1) physical measurements (self-reported height, weight, BMI), (2) a 26-item questionnaire, and (3) five items on specific behaviors in the past 6 months. This study used only the questionnaire part where scores from 0 to 78 indicate risk for eating disorders, with clinical risk suggested by scores of 20 or higher (Garner et al., 1982). The three factors the EAT-26 examines are Dieting (e.g., “I am aware of the calorie content of foods that I eat”), Bulimia and food preoccupation (e.g., “I feel that food controls my life”) and Oral control (e.g., “I avoid eating when I am hungry) (Garner et al., 1982, p. 874).

In addition to the EAT-26, the participants were asked to answer an additional 19 questions that concern the proposed four factors: Exposure to Dieting and Weight Concerns During Childhood, Importance of Food in One’s Family, Exposure to Holocaust Narratives During Childhood, and Pressure to Eat as a Child.

#### 2.2.2 Procedure

This study concerns Israelis and was conducted through an online questionnaire. The participants were gathered using convenience and snowball sampling. Participants were approached through general Facebook groups of Israelis, specific Facebook groups for Israeli descendants of Holocaust survivors, and personal communication.

### 2.3 Data analysis

Analyses were conducted using the Jamovi statistical program. An Independent Samples t-test was used to assess if 3rd-gens reported a more disordered relationship with food compared with non-3rd-gens (H1). A correlation test was run to assess the relationship between disordered eating and perception of parents’ dysfunctional relationship with food. The correlation was followed by an Independent Samples *t*-test to check difference between the 3rd-gens and non-3rd-gens (H2). To assess the difference in perceived controlling parental feeding style and attitudes toward food between 3rd-gens and non-3rd-gens, a correlation test was run and followed by an Independent Samples *t*-test (H3).

## 3 Results

Total number of respondents was 236. The youngest participants were 18 and the oldest were 50. The mean age of the participants was *M* = 27.7 and the median age was 25. The mean age of participants when split into 3d-gen and non-3rd-gen was *M* = 28.1 for 3rd-gen and *M* = 27.2 for non-3rd-gen (Table 1). Therefore, the age difference between the groups is rather balanced.

**Table 1:** Descriptives for age and EAT-26 scores split into groups.

	3rd-gen	Non-3rd gen	Group difference
*N*	130 (55.1%)	106 (44.9%)	
Mean age (standard deviation)	28.1 (7.03)	27.2 (6.55)	*t* (234) = −1.02 (*p* = 0.309)
Minimum – maximum age	18–50	18–50	
Mean EAT-26 (standard deviation)	18.0 (12.4)	16.2 (11.5)	*t* (234) = −1.14 (*p* = 0.255)

When it comes to the gender of the participants, this sample had a total of 98 men and 136 women. The total participants in the 3rd-gen group were 130, while the non-3rd-gen group had 106 participants (see Appendix Table A1).

The participants’ relationship with food was measured using the EAT-26 questionnaire (Apter and Yanko, 1989; Garner et al., 1982). Cronbach’s alpha coefficient of reliability of the questionnaire for this sample was 0.878 and McDonald’s Omega was 0.894, both of which are considered high, indicating the questionnaire showed appropriate reliability for use. However, the items ‘Other people think that I am too thin’ and ‘I display self-control around food’ correlated negatively with the total scale (see Appendix Table A2). Spivak-Lavi et al. (2023) had equivalent results regarding these two items among a sample of Jewish-Israelis. This can be due to culture differences and differences in translation. For example, the Hebrew translation for ‘Other people think that I am too thin’ would be understood as ‘Other people think that I am thin’, which can have the opposite implication as it misses the emphasis on “too thin.”

The EAT-26’s cutoff point is 20, any score above it may indicate disordered eating behaviors and attitudes (Garner et al., 1982). For this study’s sample, the mean (*M =* 17.2) and median (*Mdn =* 13.5) were below the threshold (see Table 1), with 85 of participants meeting the cut off criteria, in 3rd-gen group (*n* = 51) and in the non-3rd-gen group (*n* = 35), and there was deviation from a normal distribution according to the Shapiro–Wilk test of normality (*p* < 0.05).

The additional 19 questions confirmed the proposed four factors: Exposure to Dieting and Weight Concerns During Childhood by Parents, Importance of Food in One’s Family, Exposure to Holocaust Narratives During Childhood, and Pressure to Eat as a Child (see Appendix Table A3). The Kaiser–Mayer–Olkin (KMO) index was 0.813, exceeding the recommended value of 0.6 (Kaiser, 1970), and Bartlett’s Test of Sphericity (Bartlett, 1954) reached statistical significance [*χ*^2^(3163) = 171, *p* < 0.001]. Cronbach’s alpha coefficient of reliability of the questionnaire for this sample was 0.833, and McDonald’s Omega was 0.839, indicating high interrelatedness between the items.

To reveal if 3rd-gens reported a more disordered relationship with food compared to those with non-survivor grandparents, an Independent Samples *t*-test was used with the total scores of the EAT-26 questionnaire. Results showed that even though there was a mean difference between the groups, with 3rd-gen individuals having a higher score (*M* = 18) than the non-3rd-gen individuals (*M* = 16.2), the difference was not statistically significant, *t*(234) = −1.14, *p* = 0.255.

Nevertheless, an ANOVA test uncovered that gender significantly influenced the EAT-26 total scores, with women having more severe scores (*M =* 19.8*, Mdn =* 17) compared to men (*M =* 13.6*, Mdn =* 10) and ‘others’ (*M =* 13*, Mdn =* 13) (*p* < 0.001). This corresponds with the established claim that eating disorders and unhealthy attitudes toward food are more common among women (Bärebring et al., 2020; Striegel-Moore et al., 2009).

To check if the degree of disordered eating behaviors will be positively correlated with the participants’ perception of their parents’ dysfunctional relationship with food, a correlation test was run. The correlation matrix included the EAT-26’s total score, EAT-26’s four subscales, and the additional four subscales from the author created questionnaire. The test revealed a significant relationship between the subscale “Exposure to Dieting and Weight Concerns During Childhood by Parents” and the EAT-26’s total scores (*p* < 0.001). The correlation was positive, affirming the claim that a child’s exposure to disruptive eating and negative attitudes toward food might affect their later life development of eating disorders and problems. An Independent Samples t-test was used to determine if “Exposure to Dieting and Weight Concerns During Childhood by Parents” was stronger among 3rd-gen participants compared with non-3rd-gen participants. The results showed that although “Exposure to Dieting and Weight Concerns During Childhood by Parents” had a significant effect on the sample’s EAT-26 scores, the difference between 3rd-gen participants and non-3rd-gen participants was not significant, *t*(234) = −0.171, *p* = 0.864.

A correlation test was conducted to determine the relationship between “Exposure to Holocaust Narratives During Childhood” and “Pressure to Eat as a Child.” The test revealed a significant positive correlation (*r* = 0.374, *p* < 0.001), indicating that higher exposure to Holocaust narratives (via parents and grandparents) was associated with greater pressure to eat during childhood (experienced as a controlling feeding style by family).

To reveal if 3rd-gen participants view their parents’ feeding style and attitudes toward food as more controlling (pressured eating as a child) than non-3rd-gen participants, an Independent Samples *t*-Test with Welch’s correction was run due to a significant Levene’s test (*p* < 0.05). The test supported that 3rd-gen participants (*M* = 8.04, *SD* = 4.36) view their parents’ feeding style and attitudes toward food as more controlling than non-3rd-gen participants (*M* = 5.39, *SD* = 2.71), *t*(220) = −5.71, *p* < 0.001. The effect size (Cohen’s *d* = −0.731) suggests a medium effect.

## 4 Discussion

This study aimed to investigate whether the intergenerational transmission of Holocaust trauma from second-generation parents will manifest in their children, the third-generation, leading to unhealthy eating and attitudes toward food. Specifically we explored whether higher self-reported unhealthy eating patterns would be related to parental control around food in individuals who are considered third-generation Holocaust survivors compared to those with no such family history. The hypotheses were grounded in previous studies which, however, focused on Holocaust survivors and their children (Chesler, 2005; Kellermann, 2001c; Scharf, 2007). We therefore expanded the exploration to following generation.

The statistical analyses confirmed findings of gender differences outlined by existing literature (Bärebring et al., 2020; Striegel-Moore et al., 2009), with women reporting higher rates of disordered eating and attitudes toward food than men. In addition, the mean score for the sample was beneath the clinical cutoff point of the EAT-26 (Garner et al., 1982), in line with other studies on non-clinical populations (Doninger et al., 2005; Dotti and Lazzari, 1998; Rivas et al., 2010).

Contrary to our first hypothesis, 3rd-gens were not found to have a less healthy relationship with food than non-3rd-gens, reflected by the EAT-26 questionnaire score. We presume that while the second-generation Holocaust survivor descendants are affected in their eating habits, the effect is not generalized to the subsequent generation. Furthermore, the second hypothesis, assuming that 3rd-gens will be more exposed to dieting and weight concerns by their parents was not confirmed.

However, this study did find support for a key difference between the survivor descendants of the 3rd generation and individuals without the background: 3rd gens were statistically significantly more likely to recall their parents exerting more pressure around eating during their childhood [*t*(220) = 5.71, *p* < 0.001, Cohen’s *d* = 0.731]. The results suggest that “Controlling Parental Feeding” likely serves as a specific mediator for passing trauma to the third generation. Therefore, second-generation parents grew up hearing Holocaust stories of hunger and food shortages and thus may unconsciously push third-generation children to consume food or avoid waste, linking everyday meals to survival. This mechanism is supported in this study by the finding that higher pressure to eat was tied to exposure to Holocaust narratives as a child (*r* = 0.374, *p* < 0.001). Therefore, this daily control and dynamic concerning feeding may keep the trauma alive through family habits. This reinforces the literature fundings that parental food control, especially related to pressure to consume food, is one of the ways how trauma-related patterns are passed down to younger generation, even if the final outcome is not disordered eating in adulthood. These findings align with the study by Scharf (2007) on the intrusive and controlling feeding styles of Holocaust survivor families, echoing intergenerational trauma.

Our proposed explanation for the lack of findings of disordered eating in the 3rd gens may be related to the resilience or post-traumatic growth effects across generations (Oren et al., 2025). Furthermore, it is possible that the intensity of the eating-related trauma expression is softened in the 2-nd gen. or has been integrated in the general family culture and is therefore less pronounced in the clinical sense, lowering the scores of this group. Likewise, the recent absence of food rationing and severe food restriction (Kimhi, 2024) could be a reason why the pressure is not as strongly influential.

Practically speaking, practitioners should consider Holocaust trauma history and how it impacted the education, feeding, and current perception of eating and food of third-generation clients who report controlling feeding styles from childhood. A therapeutic method that could be effective in such cases is Family systems therapy, as it uncovers multigenerational patterns and can help rebuild healthier family food dynamics, strengthen awareness, and potentially minimize further trauma transmission to the next generations (Brown and Errington, 2024; Mooren et al., 2022; Quadrio, 2016). Another beneficial intervention is Trauma-informed nutritional counseling, which can address non-clinical food anxiety and educate about intuitive eating. Processing trauma triggers and reshaping eating attitudes and behaviors can be promoted with techniques like cognitive behavioral therapy and eye movement desensitization reprocessing (Brewerton, 2019). By understanding this issue further, appropriate interventions and therapeutic approaches can be implemented to understand better and help third-generation individuals.

### 4.1 Strengths and limitations of the current study

To the best of our knowledge, this study is the first to assess eating disorders among third-generation Holocaust survivors, addressing a gap in the literature on intergenerational trauma.

This study is not without limitations. First, the sample size of this study (*N* = 236) is relatively small. This could have affected the non-significant findings and the representation of the population and limit the generalizability of findings. Second, social factors regarding weight could be a confounding variable in the relationship between Holocaust trauma transmission and unhealthy eating and attitudes toward food. The current social norms and influences that encourage thinness might have a stronger influence on both 3rd-gens and non-3rd-gens. This possible variable was not controlled for and might be the reason for the lack of difference between the two groups, as well as the lack of additional measures of psychological distress or trauma symptoms, which may mediate eating behaviors. The lack of additional scales for anxiety or PTSD (e.g., GAD-7, PCL-5) leaves mediation between Holocaust trauma history and food attitudes partially unexplored, helping contextualize non-significant EAT-26 results despite significant parental pressure differences. Third, the study’s measures were self-reported, which can introduce bias into the data due to the participants’ recall bias. Fourth, the main instrument used in this study, the EAT-26 questionnaire, did not show optimal validity with this sample of Israeli individuals. Two items from the EAT-26 loaded very weakly in the factor analysis due to the differences in meaning and interpretation between the Hebrew translation and the original English version. Finally, the additional questionnaire constructed for this study did not go through psychometric testing; therefore, its validity and reliability (although acceptable for this sample) are unexplored.

#### 4.1.1 Recommendation for future research

Future studies should investigate the various ways in which perceived controlling feeding behaviors are manifested among third-generation individuals, their impact on these individuals’ lives, and strategies to help them cope with the adverse outcomes. Furthermore, future studies should control for modern thinness norms and incorporate validated tools like the Eating Disorder Examination Questionnaire (EDE-Q) for detailed eating psychopathology, alongside trauma inventories. Impulsivity scales, such as the UPPS-P, should be added as well. Since differences in parental pressure were observed, impulsivity may link pressure-to-eat with food attitudes. Additionally, the exploration of family trauma narrative around food, as well as its evolving nature could include elements beyond this study’s approach such as communication style within the family, identification with the cultural aspects of the trauma, and general personal anxiety levels of the respondents.

## 5 Conclusion

The data did not support the assumptions that third-generation participants would suffer from a less healthy relationship with food or that they would be more exposed to dieting and eating concerns by their parents. However, it can be deduced that third-generation participants view their parents’ feeding style and attitudes toward food as more controlling than those of non-third-generation participants, showcasing the continued transmission of trauma. Our findings highlight the importance of looking beyond the clinical thresholds and symptoms in understanding the lasting process of intergenerational trauma.

## Data Availability

The raw data supporting the conclusions of this article will be made available by the authors, without undue reservation.
