# Supplementary material for: Eating attitudes and trauma in third-generation holocaust survivors
Source: Front Psychol. 2026 May 7;17:1718777. doi: 10.3389/fpsyg.2026.1718777 (PMC13190181; doi:10.3389/fpsyg.2026.1718777)
Supplement: Supplementary file 1 [file Supplementary_file_1.pdf]

Appendices

Table A1

Frequencies of Gender split by Group (3rg-gen and non-3rd-gen)

Group	Gender	Counts	% of Total	Cumulative %
Non-3rd-gen	Man	44	18.6 %	18.6 %
	Other	2	0.8 %	19.5 %
	Woman	60	25.4 %	44.9 %
3rd-gen	Man	54	22.9 %	67.8 %
	Other	0	0.0 %	67.8 %
	Woman	76	32.2 %	100.0 %

Table A2

Component Loadings for EAT-26

	Component				
	Weight preoccupation	Concerns of others	Dieting and restricting symptoms	Binge/purge behaviors	Uniqueness
I am occupied with a desire to be thinner	0.853				0.217
I find myself preoccupied with food	0.807				0.271
I give too much time and thought to food	0.805				0.287
I engage in dieting behavior	0.785				0.219
I am terrified about being overweight	0.784				0.387

Component Loadings for EAT-26

	Component				
	Weight preoccupati on	Concerns of others	Dieting and restricting symptoms	Binge/p urge behavior s	Uniquene ss
I am preoccupied with the thought of having fat on my body	0.750				0.328
I have gone on eating binges where I feel that I may not be abl	0.704				0.557
I think about burning up calories when I exercise	0.664				0.484
I feel uncomfortable after eating sweets	0.660				0.506
I feel that food controls my life	0.658				0.463
I feel extremely guilty after eating	0.626			0.392	0.352
I display self-control around food	-0.534		0.499		0.598
I like my stomach to be empty	0.433			0.429	0.443
I feel that others would prefer if I ate more		0.855			0.243
I feel that others pressure me to eat		0.818			0.273
I take longer than others to eat my meals		0.600			0.589
Other people think that I am too thin	-0.452	0.461			0.638
I eat diet foods			0.647		0.402
I avoid foods with sugar in them			0.642		0.537
I am aware of the calorie content of foods that I eat			0.580		0.509
I particularly avoid food with a high carbohydrate content (i.e			0.531		0.443
I have the impulse to vomit after meals				0.813	0.331
I avoid eating when I am hungry.				0.623	0.350
I vomit after I have eaten				0.579	0.593
I cut my food into small pieces					0.857
I enjoy trying new rich foods-R					0.916

Note. 'promax' rotation was used

Table A3

Component Loadings for Constructed Questionnaire

	Component				
	Exposure to Holocaust	Exposure to Dieting and Weight concerns	Importance of Food	Pressure Eating	Uniqueness
Rate your level of Holocaust exposure	0.942				0.105
The Holocaust was often brought up in my family when I was growing up	0.937				0.117
I feel a strong connection to my family's Holocaust history	0.935				0.121
My parents shared memories or information about my grandparents' life at the Ghetto	0.857				0.248
My grandparents shared sad memories from the Holocaust with me while I was growing up	0.831				0.297
My grandparents shared positive memories from the Holocaust with me while I was growing up	0.820				0.318
As a child, I was often told to finish my plate because my grandparents did not have enough food during the Holocaust	0.705			0.402	0.331

Component Loadings for Constructed Questionnaire

	Component				
	Exposure to Holocaust	Exposure to Dieting and Weight concerns	Importance of Food	Pressure Eating	Uniqueness
I felt pressure to have a certain body type by my parents when growing up		0.843			0.273
One or both of my parents often talked about dieting when I was growing up		0.833			0.276
One or both of my parents often talked about their weight when I was growing up		0.797			0.328
My parents often comment about my weight		0.781			0.359
I was exposed during my childhood to terms like calories and carbs		0.729			0.466
How would you rate your parents' overall relationship with food?		-0.507			0.692
My parents stressed the importance of a healthy diet when I was growing up					0.923
It is important to my family to have a wealth of food in our house at all times			0.916		0.160
There is always plenty of food at our house			0.897		0.192

Component Loadings for Constructed Questionnaire

	Component				
	Exposure to Holocaust	Exposure to Dieting and Weight concerns	Importance of Food	Pressure Eating	Uniqueness
In my family, we always keep stocks of food			0.838		0.260
When I was growing up, it was not allowed to throw away food I				0.904	0.176
I felt pressured to finish eating my meal in my childhood				0.868	0.205

Note. 'varimax' rotation was used
